# Supplementary material for: Single-shot self-supervised object detection in microscopy
Source: Nat Commun. 2022 Dec 5;13:7492. doi: 10.1038/s41467-022-35004-y (PMC9722899; doi:10.1038/s41467-022-35004-y)
Supplement: Supplementary file 2 — Description of Additional Supplementary Files [file 41467_2022_35004_MOESM2_ESM.pdf]

Description of Additional Supplementary Files

Supplementary Movie 1: LodeSTAR successfully detects objects of various shapes, orientations and noise.

Supplementary Movie 2: Mouse hematopoietic stem cells are detected by LodeSTAR as they divide. The score map used for detection is also shown.

Supplementary Movie 3: Human hepatocarcinoma-derived cells are detected by LodeSTAR. The score map used for detection is also shown.

Supplementary Movie 4: Pancreatic stem cells are detected by LodeSTAR as they divide. The score map used for detection is also shown.

Supplementary Movie 5: Noctiluca scintillans are detected by LodeSTAR. LodeSTAR detects the optically dense area near the ventral groove where the nucleus is located. The score map used for detection is also shown.

Supplementary Movie 6: Noctiluca scintillans are detected by LodeSTAR. LodeSTAR detects the plankton as a whole. The score map used for detection is also shown.

Supplementary Movie 7: Polystyrene particles under flow are detected by LodeSTAR in 3D.

Supplementary Movie 8: SH-SY5Y cells with polysyterene particles inside and around the cell. LodeSTAR detects both the polystyrene (orange markers) and intracellular particles (blue markers).
